# Anticancer Agents Targeted to Sirtuins

**DOI:** 10.3390/molecules191220295

**Published:** 2014-12-04

**Authors:** Tomohiro Kozako, Takayoshi Suzuki, Makoto Yoshimitsu, Naomichi Arima, Shin-ichiro Honda, Shinji Soeda

**Affiliations:** 1 Department of Biochemistry, Faculty of Pharmaceutical Sciences, Fukuoka University, 8-19-1 Nanakuma, Jonan-ku, Fukuoka 814-0180, Japan; E-Mail: sihonda@fukuoka-u.ac.jp; 2 Faculty of Medicine, Kyoto Prefectural University of Medicine, 1-5 Shimogamohangi-Cho, Sakyo-Ku, Kyoto 606-0823, Japan; E-Mail: suzukit@koto.kpu-m.ac.jp; 3 Department of Hematology and Immunology, Kagoshima University Hospital, Kagoshima, 8-35-1 Sakuragaoka, Kagoshima 890-8544, Japan; E-Mails: myoshimi@m.kufm.kagoshima-u.ac.jp (M.Y.); nao@m2.kufm.kagoshima-u.ac.jp (N.A.)

**Keywords:** sirtuins, cancer, apoptosis, autophagy

## Abstract

Sirtuins are nicotinamide adenine dinucleotide^+^-dependent deacetylases of which there are seven isoforms (SIRT1–7). Sirtuin activity is linked to gene expression, lifespan extension, neurodegeneration, and age-related disorders. Numerous studies have suggested that sirtuins could be of great significance with regard to both antiaging and tumorigenesis, depending on its targets in specific signaling pathways or in specific cancers. Recent studies have identified small chemical compounds that modulate sirtuins, and these modulators have enabled a greater understanding of the biological function and molecular mechanisms of sirtuins. This review highlights the possibility of sirtuins, especially SIRT1 and SIRT2, for cancer therapy targets, and focuses on the therapeutic potential of sirtuin modulators both in cancer prevention and treatment.

## 1. Introduction

**Figure 1:**
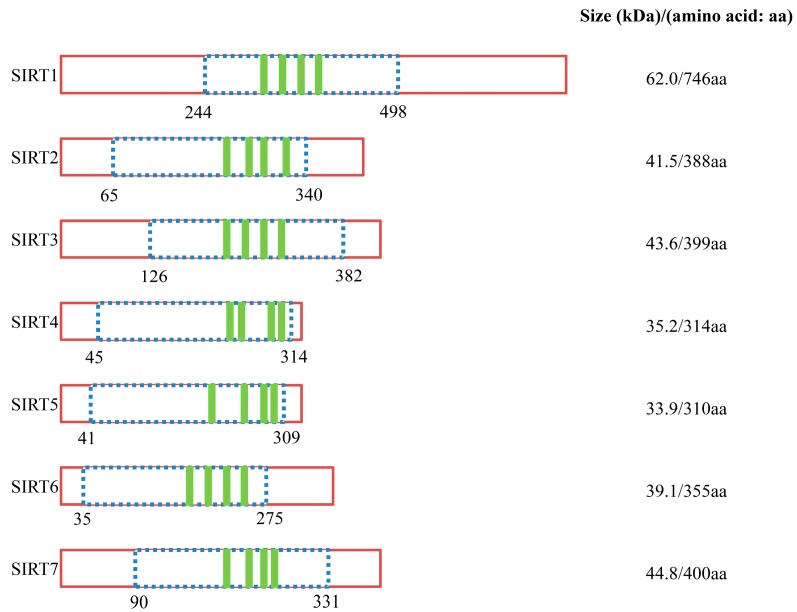
Schematic representation of human sirtuins. Sirtuins require NAD^+^ as a cofactor to exert their function with NAD^+^ dependent catalytic domain (dotted: blue) and zinc-binding domain (closed: green).

Sirtuins are members of a family of evolutionarily conserved enzymes with nicotinamide adenine dinucleotide (NAD)^+^-dependent deacetylase or mono-[ADP-ribosyl]transferase activity [1,2,3], and they can be found in nearly all species. Silencing information regulator 2 (Sir2) was the first known sirtuin identified more than 25 years ago in the budding yeast *Saccharomyces cerevisiae*. Originally discovered as a transcriptional silencer of the mating-type loci [4], many studies have demonstrated diverse biological roles for sirtuins, such as in genome stability, cellular metabolism, DNA repair, chromosomal stability, longevity and cancer [5]. Furthermore, a previous study showed that calorie or dietary restriction in rats was associated with increased levels of SIRT1, the mammalian homolog of Sir2 [6]. Mammalian sirtuins have seven isoforms (SIRT1–7), which possess primarily histone deacetylase (SIRT1, SIRT2, SIRT3, SIRT5 and SIRT7) or monoribosyltransferase activity (SIRT4 and SIRT6). Each sirtuin is characterized by an approximately 275 amino acid conserved catalytic core region and by unique additional N-terminal and/or C-terminal sequences of variable length (Figure 1) [7,8]. The N- and C-terminal regions of sirtuins are structurally disordered and have potentially interfered with crystallization efforts for the full-length proteins. However, crystal structures of the core regions of SIRT2 and SIRT5 were solved by eliminating the first 34 amino acids that were unstructured or loosely folded. These structures have a small zinc binding domain and a large Rossmann-fold domain, characteristic of NAD/NADH-binding proteins. The two domains form a space in the middle in which NAD and acetylated peptides bind [9]. Substrates of sirtuins include histones as well as other cellular proteins, including the tumor suppressor p53 (Table 1) [10]. SIRT1 and SIRT2 can be found in both the nucleus and cytoplasm. SIRT6 and SIRT7 are almost exclusively nuclear and SIRT3, SIRT4, and SIRT5 are located in the mitochondria [9]. SIRT5 also contains lysine demalonylase and desuccinylase activity [11]. Our understanding of the biology and function of sirtuins in mammalian cells has expanded significantly over the last two decades, and these enzymes have been demonstrated to play critical roles in human biology and disease [12].

**Table 1:** Diversity of sirtuins.

Class	Sirtuin	Enzyme Activity	Location	Main Substrates	High Expression	Functions
I	SIRT1	Deacetylase	Nuclear/cytoplasmic	Histone H1K26, H3K9, H3K18, H4K16, p53, FOXO, Rb, Ku70, p300, NF-κB, PGC-1α, PPARγ, UCP2, Acetyl-CoA synthetase 1, MnSOD, Smad7, MyoD, Per2, SUV39H1	Brain, skeletal muscle, heart, kidney and rterus	Cell survival, lifespan regulation, metabolism regulation, inflamation, oxidative stress response
I	SIRT2	Deacetylase	Nuclear/cytoplasmic	α-tubulin, FOXO, Histone H4K16	Brain	Cell cycle regulation, nervous system development
I	SIRT3	Deacetylase	Mitochondrial	Histone H3K9, H4K16, Acetyl-CoA synthetase 2, Ku70, FOXO3a, MnSOD	Brain, heat, liver, kidney and brown adipose tissue	Regulation of mitochondrial metabolism, fatty acid oxidation, ATP-production
II	SIRT4	ADP-ribosyl-transferase	Mitochondrial	Glutamate dehydrogenase	Pancreatic b-cellls, brain, liver, kidney and heart	Regulation of mitochondrial metabolism, insulin secretion
III	SIRT5	Deacetylase/Demalonylase/Desuccinylase	Mitochondrial	Carbamoyl phospate synthetase 1, Cytochrome c, Carbamoyl phosphate synthetase 1	Brain, testis, heart, muscle and lymphoblast	Apoptosis, regulation of protein-protein interactiond and protein stability, urea cycle
IV	SIRT6	Deacetylase/ADP-ribosyl-transferase	Nuclear	Histone H3K9, H3K56, TNF-α, HIF1α	Brain, muscle, heart, ovary and bone cells (absent in bone marrow)	Genome stability (base excision repair), DNA rapair
IV	SIRT7	Deacetylase	Nuclear	Histone H3K18, p53	Peripheral blood cells, CD33+ myeloid bone marrow precuseor cells	Regulation of rRNA transcription, cell cycle regulation

Recently, a great deal of research has focused on the identification of small chemical compounds that modulate sirtuins [13,14]. This review presents the most significant findings of sirtuins with an emphasis on SIRT1 and SIRT2 as small-molecule modulators of SIRT1 and SIRT2 and their therapeutic potential against cancer.

## 2. Sirtuins in Cancer

SIRT1 can activate stress defense and DNA repair mechanisms, and therefore aids in the preservation of genomic integrity [5]. SIRT1 also functions in the regulation of metabolism and maintaining the integrity of the genome, and has thus been described as a potential tumor suppressor. Notably, both breast cancer and hepatic cell carcinoma exhibit reduced SIRT1 levels compared with normal tissues [15]. SIRT1 also may have suppressive activity in tumor cell growth by suppressing NF-κB [16,17], a transcription factor that plays a central role in the regulation of innate and adaptive immune responses and carcinogenesis. The dysregulation of NF-κB can lead to the onset of tumorigenesis and tumor malignancy [18]. Another important role for SIRT1 in cancer is its suppression of the apoptosis inhibitor survivin in breast cancer susceptibility gene 1 (BRCA1)-associated breast cancers [19]. SIRT1 mRNA and protein levels were significantly lower in cell lines derived from mammary tumors lacking BRCA1 compared with cell lines derived from tumors without BRCA1 mutation. Furthermore, SIRT1 activity is required for suppressing survivin transcription. BRCA1 binds to the SIRT1 promoter and increases SIRT1 expression, which in turn inhibits survivin by changing the epigenetic modification of histone H3 [19]. Thus, reduction of survivin via SIRT1 activity may play an important role in BRCA1-associated mammary tumor formation.

Conversely, other studies showed that overexpression of SIRT1 caused the suppression of DNA damage repair proteins and factors involved in tumor suppression, and led to increased tumor growth and cell survival [20]. For example, SIRT1 is upregulated in many different cancers, including lymphomas, leukemia and soft tissue sarcomas, prostate cancer, lung cancer, and colon carcinomas [21,22]. When tumor suppressor genes are inactivated, resulting in a loss or reduction of their function, the cells can progress to cancer. SIRT1 has been proposed to promote tumor development and progression, as SIRT1-mediated deacetylation suppresses the functions of several tumor suppressors, including p53, p73, and hypermethylated in cancer 1 (HIC1) [23,24,25]. SIRT1 inhibition (by small molecule SIRT1 inhibitors or SIRT1 knockdown) induces cell death via apoptosis and acetylation of p53 and p73 [12,22,24]. However, the SIRT1 inhibitor nicotinamide could not restore HIC1 expression in lung cancer cells [26]. A previous study showed that SIRT1 binds p53 and deacetylates its C-terminal Lys382, resulting in inhibition of p53 induction of cell cycle arrest and apoptosis in response to DNA damage and oxidative stress [27]. From these data, SIRT1 has been suggested to exhibit tumor-promoting functions in the cell. Interestingly, SIRT1 interacts with HIC1, a tumor suppressor and transcriptional repressor that is epigenetically inactivated but not mutated in human cancers [28]. SIRT1 and HIC1 form a transcriptional repression complex that directly binds the SIRT1 promoter and represses SIRT1 transcription. Depletion of HIC1 expression results in increased SIRT1 level. This results in the deacetylation of p53, which attenuates its function. SIRT-mediated inhibition of p53 blocks p53-mediated apoptosis and DNA damage signaling, and thus contributes to tumor development. In addition, silencing of the HIC1 promoter by hypermethylation during aging results in an upregulation of SIRT1. This enhancement of SIRT1 in aging cells may increase cell survival as well as cancer risk [25]. Thus, SIRT1 could act as either a tumor suppressor or tumor promoter, depending on the cellular context or its targets in specific signaling pathways or specific cancers. However, the precise mechanisms underlying these contradictory activities are not well understood.

SIRT2 is a tumor suppressor gene that has an essential role in maintaining the integrity of mitosis by positively regulating the activity of anaphase-promoting complex/cyclosome. Its dysfunction leads to genetic instability and tumorigenesis [29]. During mitosis, SIRT2 is relocalized from the cytoplasm to the nucleus and serves as a histone deacetylase with a preference for histone H4 lysine 16 [30]. Overexpression of SIRT2 can significantly prolong the mitotic phase and delay mitotic exit. Thus, it has been proposed that SIRT2 might function as a mitotic checkpoint protein in G2-M to prevent the induction of chromosomal instability, particularly in response to microtubule inhibitor-mediated mitotic stress [31]. Several studies have shown that tumors that express high levels of SIRT2 are not responsive to chemotherapy, specifically microtubule poisons [32]. Human SIRT2 is most predominantly expressed in the brain [33] and SIRT2 mRNA expression is severely reduced in approximately 70% of human gliomas [34]. While SIRT2 inhibition can reduce α-synuclein-induced cytotoxicity in cellular and *Drosophila* models of Parkinson’s disease [35], decreased SIRT2 activity can lead to apoptosis of C6 glioma cells [36]. SIRT2 reductions can also induce apoptosis of HeLa cells by affecting the levels of p53 [37].

Recently, hypoacetylation of histone H3 acetyl lysine 18 (H3K18Ac) has been reported to be a general marker of tumor prognosis and oncoviral transformation [38]. H3K18Ac has also been linked to tumorigenesis, as well as poor prognosis and aggressive tumor phenotypes [39,40]. Previous studies showed that SIRT7 binds specific promoters and deacetylates H3K18Ac, causing repression of transcription. SIRT1 is also responsible for site-specific deacetylation at H3K18Ac in cancer cells [41]. SIRT7 plays a critical function in maintaining properties of cancer cells, including escape from cell contact inhibition and anchorage-independent growth. Adenovirus E1A induction of malignant cell transformation involves global hypoacetylation of H3K18Ac, and SIRT7 is also essential in this process. Furthermore, human cancer cell xenografts that lack SIRT7 exhibit markedly reduced oncogenicity in mice. Thus, SIRT7 is a highly selective H3K18Ac deacetylase and has a pivotal role in chromatin regulation, cellular transformation, and tumor formation [38].

Expression of various sirtuins is altered in many types of cancers (Table 1). For example, SIRT1, 4, 5, and 7 have been described as being upregulated in certain cancers [42,43,44,45,46], while reduced SIRT1 levels have been reported in breast cancer and hepatic cell carcinoma [15]. SIRT2 is downregulated in gliomas and gastric carcinomas [47], as well as in melanomas, in which a mutation in its catalytic domain has been shown to eliminate its enzymatic activity [48]. SIRT6 is also downregulated in pancreatic cancer and colon adenocarcinoma [49]. The case of SIRT3 is more complex since it has been found to be upregulated or downregulated in different types of breast cancers [50]. SIRT7 knockdown in human cells induces cell cycle arrest and apoptosis [51,52]. Some sirtuins, such as SIRT2 and SIRT6, seem to function as tumor suppressors, but others, such as SIRT1, are apparently bifunctional, operating as both tumor suppressors and oncogenic factors, depending on cellular context and study conditions. Recent findings have suggested that these contradictory activities of sirtuins might actually be a “double-edged sword”; however the mechanisms underlying these functions remain unknown.

## 3. Sirtuins and Cell Death

For the maintenance of homeostasis, regulated cell death plays a key role in a variety of biological processes including tissue sculpting during embryogenesis, development of immunity, and destruction of damaged cells and tumors [53]. Apoptosis and necrosis are the two major modes of cell death [54]. Recently, autophagy, which is a predominantly cytoprotective process that can degrade cellular components independently of caspase activity, has been linked to both types of cell death, serving either a pro-survival function or a pro-death function [55,56,57,58]. Autophagy and necroptosis (which is a programmed form of necrotic cell death and caspase-independent cell death induced by death receptors [59]) are intricately linked processes. Furthermore, a previous study showed that sirtuins could influence apoptosis and autophagy [5,60].

Based on cell-culture models, many studies have shown that SIRT1 can inhibit apoptosis and senescence [61,62,63], suggesting that SIRT1 inhibition may be beneficial for treating certain types of cancers [64]. The most important function of activated p53 is to induce cell cycle arrest, apoptosis, and DNA repair, as mentioned previously. SIRT1 has been demonstrated to reduce p53-mediated apoptosis [61] and negatively regulate p53-induced cellular senescence [65]. In addition, more than half of all human cancers are related to p53 mutations, and a strong body of evidence suggests that cancers in which p53 is not mutated exhibit some alteration in its pathway [66]. Previous studies demonstrated that SIRT1 regulates both p53 transcription-dependent and p53 transcription-independent apoptosis pathways [23,67]. SIRT1 regulates p53 in various ways, chiefly via deacetylation of p53, which induces inactivation of p53 and inhibition of p53-dependent apoptosis [27]. Another mechanism by which SIRT1 regulates p53 is by affecting p53 subcellular localization, as part of the mitochondrial-dependent apoptotic response [68]. When intracellular reactive oxygen species are high, SIRT1 deacetylates p53 and blocks its nuclear translocation, leading to the accumulation of p53 in both the cytosol and mitochondria. This subsequently results in transcription-independent p53-induced apoptosis. Therefore, inhibition of SIRT1 activity that leads to elevated p53 acetylation and transactivation, and results in enhanced apoptosis and cytostasis, would be beneficial for cancer treatment. Furthermore, SIRT1 is involved in the regulation of the retinoblastoma (Rb) tumor suppressor, which interacts with E2F to regulate the cell cycle [69]. The activity of Rb is regulated by phosphorylation and acetylation at multiple residues. Formation of the Rb-SIRT1 complex results in deacetylation of Rb by SIRT1, leading to inhibition of Rb-dependent apoptosis [70]. Sirtuins also regulate other critical apoptotic factors, including the forkhead-box (FOXO) transcription factors, which are very important in both stress response and cancer because of their roles in cell cycle arrest, DNA repair, and apoptosis [71]. FOXO proteins, fusion proteins that result from chromosomal translocations in various cancers, are tumor suppressors [72,73]. FOXO protein is deacetylated by SIRT1, resulting in enhanced transcription of FOXO target genes that function in stress resistance and decreased transcription of apoptosis-related genes [62]. However, SIRT1 directly interacts with the N-terminus of the signaling protein Smad7, which induces tumorigenicity by blocking TGF-β-induced growth inhibition and apoptosis. This reverses p300-mediated acetylation of 2 lysine residues (Lys-64 and Lys-70) on Smad7. Overexpression of SIRT1 leads to a reduction of Smad7 levels in mesangial cells and attenuates both Smad7- and TGF-β-induced mesangial cell apoptosis, whereas SIRT1 knockdown enhances apoptosis [74]. Additional biochemical studies have revealed that the deacetylation of Smad7 by SIRT1 promotes its ubiquitination, enhances Smad ubiquitination regulatory factor 1-mediated degradation, and thereby inhibits TGF-β-dependent apoptosis [74].

Thus, these data demonstrate that the effects of SIRT1 overexpression in both increasing cell growth and blocking apoptosis would promote tumor formation and development. However, sirtuins are not always committed to cell survival. Under certain extreme conditions, such as chronic stress, SIRT1, SIRT2, and SIRT3 can protect the organism by inducing cell senescence or apoptosis [75,76,77].

Accumulating evidence implicates a function for sirtuins in autophagy [5,56,60,78]. Indeed, SIRT1 forms a complex with several autophagy components, such as ATG5, ATG7 and ATG8, and deacetylates them to induce autophagy [60]. In addition, the FOXO1 transcription factor has been shown to dissociate from SIRT2 in human cancer cells in response to oxidative stress or starvation. In turn, FOXO1 becomes acetylated and binds to ATG7, inducing autophagy [79]. Thus, both SIRT1 and SIRT2 function and respond to various nutrient stress conditions to modulate autophagy.

## 4. Sirtuin Inhibitors

Sirtuins play a key role in several pathologies. Recently, multiple research groups have pursued the identification and development of small-molecule compounds that modulate sirtuins [80]. While the beneficial impact of increased SIRT1 activity observed in several animal models favored the discovery and design of pharmacological activators of sirtuins, presumably as a calorie restriction mimetic, sirtuin inhibitors can also be potentially useful as therapeutic agents because upregulated SIRT1 has been described in cancers [21,22]. This raises the possibility that SIRT1 inhibition might suppress cancer cell proliferation. In recent years a number of inhibitors have been discovered and characterized, including splitomicin and its analogs, the indole derivative EX-527, sirtinol, salermide, cambinol, tenovins, suramin, AGK2, and others (Table 2).

A screen for inhibitors of yeast Sir2 led to the identification of splitomicin and its analogues [80]. Although splitomicin did not act efficiently against human sirtuins, several analogs with a different orientation of the β-phenyl group were developed and characterized for their activity on SIRT2 [81]. Splitomicin derivatives as SIRT2 inhibitors show antiproliferative properties and cause tubulin hyperacetylation in MCF7 breast cancer cells. These derivatives are promising candidates for further optimization as potential anticancer drugs [82].

EX-527 is a cell-permeable, selective inhibitor of SIRT1 (IC_50_ = 98 nM). Treatment with EX-527 dramatically increases acetylation at lysine 382 of p53, which has been shown to correlate with p53 activation after DNA damage induction in primary human mammary epithelial cells and several other cell lines [83]. However, it has no effect on the expression of p53 target genes, cell viability, and proliferation in various tumor lines [83]. Interestingly, sirtinol and salermide, which are SIRT1/2 inhibitors, can induce p53 acetylation at lysine 382, but EX527 does not do the same in MCF-7 cells [12]. EX527 is ineffective in inhibiting SIRT2. However, p53 mediates the cytotoxic function of sirtinol and salermide. Studies using breast carcinoma cell lines and p53-deficient mouse fibroblasts confirmed that p53 is essential for sirtinol and salermide-induced apoptosis. SIRT inhibitors require combined targeting of both SIRT1 and SIRT2 to induce p53 acetylation and cell death.

**Table 2:** Sirtuin inhibitors.

Inhibitors	Structure	MW	Target (IC_50_)
Splitomicin	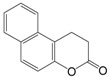	198.22	Sir2 (60 μM)
EX-527	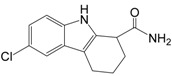	248.71	SIRT1 (98 nM)
Sirtinol	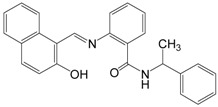	394.47	SIRT1 (131 μM),
			SIRT2 (49 μM)
Cambinol	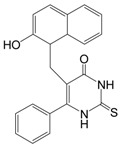	360.43	SIRT1 (56 μM),
			SIRT2 (59 μM)
Salermide	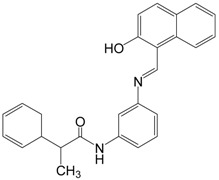	394.5	SIRT1 (76.2 μM),
			SIRT2 (45 μM)
Tenovin-6	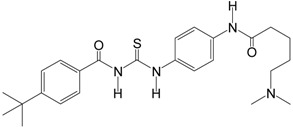	454.6	SIRT1 (21 μM),
			SIRT2 (10 μM),
			SIRT3 (67 μM)
Suramin	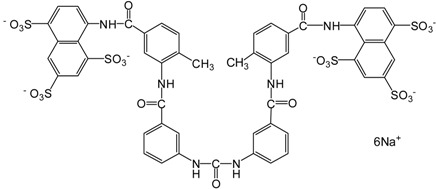	1429.17	SIRT1 (297 nM),
			SIRT2 (1.2 μM),
			SIRT5 (22 μM)
AGK2	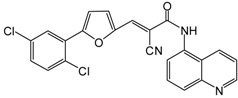	434.27	SIRT2 (3.5 μM)

Sirtinol, with the chemical name 2-[(2-hydroxy-naphthalen-1-ylmethylene)-amino]-N-(1-phenyl-ethyl)benzamide, inhibits both yeast Sir2, human SIRT1 and human SIRT2 activity* in vitro*. The 2-hydroxyl-1-napthol moiety was shown to be sufficient for inhibition [84]. Sirtinol induces senescence-like growth arrest in human breast cancer MCF-7 cells, lung cancer H1299 cells [85], and enhances chemosensitivity to camptothecin and cisplatin in DU145, PC3, and HeLa cells, leading to enhanced apoptosis and a subsequent significant cell growth inhibition [12,86]. Sirtinol also induced significant growth inhibition or apoptosis in cells from adult T-cell leukemia-lymphoma (ATL) that develops after long-term infection with human T-cell leukemia virus (HTLV-1) and leukemic cell lines, especially HTLV-1-related cell lines [22]. Sirtinol-induced apoptosis was mediated by activation of the caspase family and degradation of SIRT1 in the nucleus. Furthermore, SIRT1-knockdown by SIRT1-specific small interfering RNA caused apoptosis via activation of caspase-3 and PARP in an HTLV-1-related cell line. Interestingly, sirtinol decreases the expression of SIRT1 in MCF-7 breast cancer cells, causing cell cycle arrest in the G1 phase and apoptotic cell death, while inducing caspase-independent autophagic cell death in MCF-7 cells [87]. Sirtinol also induces autophagic cell death in THP-1 cells, which is a human monocytic cell line derived from an acute monocytic leukemia patient [88].

Cambinol and salermide are reported as inhibitors of SIRT1 and SIRT2 [89,90]. Cambinol is a cell-permeable β-naphthol compound that inhibits the NAD-dependent deacetylase activity of SIRT1 and SIRT2 (IC_50_ = 56 μM and 59 μM, respectively) in a substrate-competitive but not NAD-competitive manner [89]. Cambinol induces BCL6 acetylation and exhibits the most potent effects in Burkitt lymphoma cell lines [89]. Salermide induces apoptosis in cancer cells in a p53-independent manner [12].

Tenovin-1, along with a more water-soluble analog, tenovin-6, is able to inhibit SIRT1/2 at single-digit micromolar concentrations and prevent tumor growth* in vivo* by p53 activation [91]. Tenovin-6 inhibits the protein deacetylase activities of purified human SIRT1, SIRT2, and SIRT3* in vitro* with IC_50_ values of 21, 10, and 67 µM, respectively, and induces apoptosis in gastric cancer via death receptor 5 [92]. Tenovin-6 also increases autophagosome and consistently dysregulates autophagy, while tenovin-6 does not induce cellular apoptosis or p53-pathway activity in chronic lymphocytic leukemia [93]. Suramin is also a potent inhibitor of many sirtuins. It inhibits SIRT1 with an IC_50_ of 297 nM, SIRT2 with IC_50_ of 1.15 μM, and SIRT5 with an IC_50_ of 22 μM. However, its neurotoxicity limits its therapeutic use [94]. These observations further indicate that dual SIRT1/2 inhibition or broad-spectrum sirtuin inhibitors may provide therapeutic benefits in cancer. However, the efficacy and safety of these inhibitors need to be fully assessed.

Another potent and selective inhibitor of SIRT2 is AGK2 with an IC_50_ of 3.5 μM. It was demonstrated that the inhibition of SIRT2 by AGK2 protects against dopaminergic cell death in a *Drosophila* model of Parkinson’s disease [35]. Interestingly, AGK2 induces caspase-3-dependent apoptosis and necrosis of C6 glioma cells [36], while SIRT2 is downregulated in gliomas [34]. Thus, inhibition of SIRT2 may become a new strategy for treating gliomas.

We have demonstrated that novel small molecule SIRT1 inhibitors reduced cell viability and enhanced apoptosis in peripheral blood mononuclear cells of patients with acute ATL, which has a poor prognosis (manuscript submitted). Interestingly, these inhibitors also reduced the cell viability in caspase-dependent or -independent manners in leukemic cell lines.

Furthermore, other selective or multiple inhibitors of sirtuins, such as amurensin G, UVI5008, and JBG1741, have been described [95,96,97,98,99,100]. Interestingly, acetylation of histone H3K18Ac is particularly important, because specific deacetylation of H3K18Ac is indispensable for oncogenic transformation by adenovirus [101] and for host responses to bacterial infection [102]. Regarding the former, it has also been demonstrated that H3K18Ac hypoacetylation is linked to the maintenance of malignant phenotypes [38] and poor prognosis [39] in cancer. A recent study indicated that alkylating agents induce histone H3K18Ac hyperacetylation through SIRT1 and SIRT7 downregulation and histone deacetylase 3 cleavage [41].

## 5. Sirtuin Activators

Studies have shown that the sirtuin activator resveratrol, a polyphenol found in wines and thought to harbor major health benefits, induces apoptosis in response to TNFα via NF-κB inhibition, and has chemopreventive activity against various cancers, including leukemia, skin cancer, and prostate cancer [17,103,104,105,106]. Resveratrol also induces autophagy, and it has been shown that this occurs in a SIRT1-dependent manner [107]. On the other hand, in a cell-culture model of rotenone-induced cell death, resveratrol was reported to protect against rotenone-induced apoptosis and enhance degradation of α-synucleins, which was shown to occur by induction of autophagy [108]. The resveratrol derivative Longevinex has the curious effect of increasing autophagy after prolonged administration, and this correlates with increased SIRT1 and SIRT3 levels, as well as FOXO nuclear translocation [109]. Other phenol derivatives including quercetin and piceatannol were also shown to have SIRT1-activating properties [110].

Current studies in animal models are examining the biological functions of SIRT1 activators with the aim of identifying cancer treatments [80]. Resveratrol has been used extensively as a tool to induce SIRT1 activity in cells and* in vivo* [111]. SRT1720, SRT2183, and SRT1460 were recently described as SIRT1 activators [112]. They are structurally unrelated to resveratrol and were reported to activate SIRT1 with potencies 1000-fold greater than resveratrol. SRT1720 was found to inhibit growth and induce apoptosis in multiple myeloma cells with overexpression of SIRT1 that are resistant to conventional and bortezomib therapies without significantly affecting the viability of normal cells [113]. SRT1720 also inhibits multiple myeloma tumor growth in animal tumor studies. However, its activity is still debated [114]. SRT1720, SRT2183, SRT1460, and resveratrol are not direct activators of SIRT1. In the literature, resveratrol has been widely referred to as a SIRT1 activator [80] and is routinely used to activate SIRT1 in various cellular assays. A few researchers questioned the reported ability of activating SIRT1 in an artificial substrate-based fluorescent assay [115,116]. Given that the activation of SIRT1 by resveratrol requires the use of peptides conjugated with a non-physiological fluorophore, and that no activation was observed when peptides lacking this fluorophore were used, other technical approaches are necessary to establish its effective modulation of SIRT1 [115].

There have been conflicting data reported in the literature that support both activation and inhibition of SIRT1 as a strategy for cancer therapy. SIRT1 inhibitors and SIRT1 activators can contribute anti-tumor effects by promoting apoptosis and inhibiting cell growth in cancer with aberrant overexpression of SIRT1, although the precise mechanisms underlying these contradictory activities are not well elucidated. Thus, sirtuin modulators may provide therapeutic benefit in cancer and require further investigation.

## 6. Conclusions

There is evidence that sirtuin modulation can be beneficial for a wide variety of diseases, including cancer. Thus, the functions of SIRT1 in stimulating cell growth and angiogenesis, and blocking senescence and apoptosis indicate that SIRT1 may play a critical function in tumor initiation, progression, and drug resistance. However, there is still a long way to go for treating SIRT1-related diseases such as cancer. The role of the other sirtuins in cancer remains to be further clarified. The precise biological pathways by which sirtuins affect the cell cycle, immune system, and cellular metabolism are areas of intense study. Sirtuin modulators have shown promising anticancer effects in animal models of cancer. Thus, new approaches with sirtuin inhibitors and activators may provide different prophylactic and therapeutic strategies for cancer treatment.

## Abbreviations

ATG: autophagy-related protein
ATL: adult T-cell leukemia-lymphoma
BRCA1: breast cancer susceptibility gene 1
FOXO: forkhead-box
H3K18Ac: H3 acetyl lysine 18
HIC1: hypermethylated in cancer 1
HTLV-1: human T-cell leukemia virus
IC_50_: 50% inhibitory concentration
NAD: nicotinamide adenine dinucleotide
NF-κB: nuclear factor-κB
Rb: retinoblastoma
RIP3: receptor-interacting protein kinase 3
Sir2: Silencing information regulator 2
TGF-β: transforming growth factor-β
TNF-α: tumor necrosis factor-α
